# Supplementary material for: Learning curves for itinerant nurses to master the operation skill of Ti-robot-assisted spinal surgery equipment by CUSUM analysis: A pilot study
Source: PLoS One. 2024 Mar 11;19(3):e0291147. doi: 10.1371/journal.pone.0291147 (PMC10927145; doi:10.1371/journal.pone.0291147)
Supplement: Attachment — Submitted filename: Response to Reviewers.docx [file pone.0291147.s002.docx]

**Dear Editor and Reviewers:**

We are very glad to receive your reply.On behalf of my co-authors ,we thank you very much for giving us an opportunity to revise our manuscript again ,we appreciate editor and reviewers very much for their positive and constructive comments and suggestions on our manuscript entitled:“**learning curves for itinerant nurses to master the operation skill of Ti-robot assisted spinal surgery equipment by CUSUM analysis**”.（PONE-D-23-11364R1）.

We have studied comments carefully and have made correction which we hope meet with approval. Revised portion are marked in red in the paper. The main corrections in the paper and the responds to the editor and reviewer’s comments are as following:

**Dear Editor priti chaudhary:**

**Responds to the editor’s comments:**

**1. Authors are requested to write Funding information in Financial Disclosure column (as per authors-"The research was supported by the Baoding First Central Hospital ,Hebei province,China.")**

**Response:** Thank you for your notifications.We have added Funding information in the corresponding sections.But we have some questions about why this part is not displayed in the generated PDF file? Looking forward to your reply.

**2. As the sample size is small, study may be considered as Pilot project/ study.**

**Response :**Thank you for your suggestion.We think this is a very good proposal.We have revised the title of the article.The revised title is **“Learning curves for itinerant nurses to master the operation skill of Ti-robot-assisted spinal surgery equipment by CUSUM analysis：A pilot study”**

In addition, we added a co first author Huiyue Wang, who contributed substantially to the statistical portion of the study.

Following the request of the journal, we modified the format of the references.

According to your prompts, we have uploaded our figure files to the PACE digital diagnostic tool, ensured that figures meet PLOS requirements.

Thanks again to all editors and reviewers for their comments on revisions. We look forward to hearing from you.

Best wishes

Yichao Yao August 5, 2023
